# Understanding functional diversity in public primary health care: a cluster analysis of utilization patterns

**DOI:** 10.3389/frhs.2026.1782809

**Published:** 2026-05-15

**Authors:** Stefanos Karakolias, George Pontisidis, Nikolaos Polyzos

**Affiliations:** 1 Department of Nursing, Democritus University of Thrace, Alexandroupolis, Greece; 2 Department of Medicine, Democritus University of Thrace, Alexandroupolis, Greece

**Keywords:** case-mix, cluster analysis, functional diversity, geographical location, primary health care, service utilization, unit size, unit type

## Abstract

**Background:**

Primary health care (PHC) systems are typically organized using uniform administrative models that assume functional similarity across facilities. In practice, however, PHC units differ substantially in how services are delivered. In Greece, despite ongoing reforms, empirical evidence on the functional heterogeneity of public PHC units remains limited. This study aims to classify PHC units based on observed service utilization patterns and to identify the organizational and geographical determinants shaping these functional typologies.

**Methods:**

This cross-sectional study analyzed administrative data from 649 public PHC units in Greece (2024). Visit compositions (regular, emergency, prescription, and other) were analyzed using compositional data analysis with centered log-ratio transformation. K-means clustering was applied to identify utilization-based functional typologies. Associations between cluster membership and unit characteristics (size, organizational type, and geography) were examined using ANOVA and chi-square tests.

**Results:**

Three distinct functional typologies emerged. Cluster 1 (“balanced, generalist units”, *n* = 308) exhibited a mixed service profile across visit types. Cluster 2 (“routine care–focused units”, *n* = 66) was dominated by regular visits (82.4%) and minimal prescription activity, but showed substantial within-cluster variability in emergency care. Cluster 3 (“mixed, higher-intensity units”, *n* = 275) displayed a heterogeneous case-mix and a greater share of complex or administrative activity. Cluster membership was significantly associated with unit size, organizational type, and geographical location (*p* < 0.001). Cluster 1 units were significantly larger, followed by Cluster 3, while Cluster 2 units were the smallest. Organizationally, Cluster 1 was dominated by Rural Health Centers and Local Health Units, Cluster 2 by Local Medical Practices and Urban Health Centers, and Cluster 3 by Multipurpose Regional Medical Practices and Local Health Units. Spatially, higher-intensity units (Cluster 3) were predominantly located in major urban centers.

**Conclusion:**

PHC is functionally heterogeneous, with distinct utilization profiles that are not captured by existing administrative classifications. These findings reveal a structural misalignment between formal organizational labels and actual service delivery. A utilization-based typology provides a practical tool for health policy and management, supporting more targeted approaches to resource allocation, workforce planning, and regional service design. Incorporating functional differentiation into PHC governance can enhance efficiency, equity, and system responsiveness.

## 1 Introduction

### 1.1 Conceptual framework

Primary health care (PHC) is a central pillar of health system performance, with strong PHC systems associated with improved population health outcomes, reduced inequalities, and more efficient use of resources (1). From a health policy and management perspective, PHC plays a critical role in prevention, chronic disease management, coordination of care, and the regulation of patient flows across health system levels.

Despite its importance, PHC systems are often managed using uniform organizational models that assume functional similarity across facilities. In practice, however, PHC units differ substantially in how they allocate effort across regular consultations, urgent care, prescription-related encounters, and other services. These differences shape workload, staffing needs, costs, and patient access, making functional diversity a key but underexplored dimension of PHC governance.

PHC units typically provide general medical care, preventive services, maternal and child health services, and chronic disease management (2). In many systems, they also integrate mental health services (3), act as gatekeepers to specialized care (4), and manage urgent but non-life-threatening conditions (5). Prior research has described broad functional orientations, including units focused on routine care and continuity (6–9), urgent or emergency care (10–14), and prescription-related services (15, 16). However, these classifications are usually inferred from organizational type or service mandates rather than from observed service utilization.

From a management perspective, reliance on administrative labels alone risks obscuring substantial within-category variation. PHC units frequently combine multiple service roles, and their operational profiles evolve in response to local demand, workforce availability, and geographic constraints (17, 18). As a result, facilities with the same formal designation may require different staffing models, funding mechanisms, and performance benchmarks. Yet, empirical, utilization-based classifications of PHC units at the national level remain scarce.

Taken together, understanding functional typologies is essential for designing PHC systems that are efficient, equitable, and responsive to population needs, and for informing policy and planning decisions aimed at improving service delivery and system performance.

### 1.2 The Greek case

Greece offers a particularly informative case for examining functional diversity in PHC within a policy and management context. Its public PHC system operates within a mixed public–private environment and has undergone successive reforms shaped by fiscal austerity, decentralization, and rising demand linked to demographic change and migration (19). These reforms have aimed to strengthen comprehensive, community-based PHC while improving access and coordination across regions.

Public PHC provision in Greece is organized through a decentralized network of facilities, including urban and rural Health Centers, and various regional and local medical practices (20). Although these units are formally embedded in a unified national framework, they operate under markedly different geographic, demographic, and organizational conditions. Existing evaluations emphasize generally positive patient experiences with medical care and communication, alongside persistent challenges related to waiting times, preventive services, and person-centered care (21, 22).

However, evidence on Greek PHC has focused primarily on patient satisfaction and regional service availability, offering limited insight into how PHC units differ in their functional roles. For policymakers and managers, this represents a critical evidence gap, as effective PHC planning depends not only on where facilities are located or how they are labeled, but on how they actually function in practice.

### 1.3 Study contribution

This study addresses this gap by adopting a data-driven, utilization-based approach to classifying public PHC units in Greece. Using compositional data analysis and cluster analysis, we identify empirically derived functional typologies based on observed case-mix patterns, rather than assumed roles derived from organizational categories.

From a health policy and management perspective, this approach enables a more realistic assessment of PHC system organization and performance. Greece provides a theoretically relevant setting because it combines centralized regulation with pronounced regional and organizational heterogeneity, allowing functional differentiation to be examined within a formally unified system. The methodological framework is readily replicable in other health systems using routine administrative data.

The study makes three main contributions. First, it provides the first nationwide empirical classification of Greek public PHC units based on service utilization patterns. Second, it demonstrates how compositional methods can support evidence-informed PHC governance by revealing hidden functional heterogeneity. Third, it links functional typologies to organizational and geographic determinants, providing policymakers with actionable tools for aligning financing, workforce planning, and service design with actual patterns of care delivery.

## 2 Methods

### 2.1 Study design

This study employed a cross-sectional observational design based on administrative data capturing the activity of all public PHC units in Greece. The analysis pursued two objectives: first, to identify functional typologies of PHC units based on the relative composition of visit types; and second, to examine whether cluster membership was associated with unit size, organizational type, and geographical location.

### 2.2 Data source and study population

Data were obtained from the official annual report of the Hellenic Ministry of Health (MoH) entitled “Activity of Primary Health Care Units, 2024” (23). The original dataset included 653 public PHC units. Four units with zero recorded visits across all categories were excluded as non-operational, resulting in a final analytic sample of 649 units representing the full spectrum of public primary care provision in Greece.

Additionally, aggregated population data from the Hellenic Statistical Authority (ELSTAT) were used, providing estimated populations for each regional unit as of January 1, 2024 (24). These data supported the calculation of two indices: the PHC Unit Density (PHCUD) which reflects the number of PHC units per 100,000 inhabitants, and the Population-Adjusted Visit Rate (PAVR), expressing the average visits per inhabitant by visit type. Because catchment areas and populations of individual PHC units are not clearly defined, both indices were used for descriptive purposes only and were excluded from the cluster analysis, which was based solely on unit-level visit data.

### 2.3 Data preparation and variable construction

For each PHC unit *i*, the visit composition was defined by the proportion vector.

$$p_{i}=(\;p_{i,r},p_{i,e},p_{i,p},p_{i,o})$$

where each component denotes the share of visits of a specific type, namely regular (r), emergency (e), prescription (p), or other (o), with respect to the total visits recorded at that unit.

The category “other visits” corresponds to a residual administrative classification used in the MoH reporting system and includes encounters that do not fall under regular consultations, emergency visits, or prescription-related services. These may comprise administrative encounters, preventive or follow-up visits not otherwise specified, referrals, or service-specific activities recorded outside standard visit categories. Because this category is heterogeneous by definition, it was treated analytically as a distinct but non-specific component of the visit composition.

Because visit proportions are constrained to sum to one, the data are inherently compositional. Units with zero values for specific visit types were adjusted by adding a small pseudo-count (+0.5) prior to transformation, following established practice in compositional data analysis (CoDA), to avoid undefined logarithmic transformations (25). Sensitivity analyses were performed and verified the robustness of results to the choice of pseudo-count (26).

The compositional vectors were subsequently transformed using the centered log-ratio (clr) transformation, defined as

$$clr(\;p_{i})=\left(\ln\frac{\;p_{i,r}}{g(\;p_{i})},\ln\frac{\;p_{i,e}}{g(\;p_{i})},\ln\frac{\;p_{i,p}}{g(\;p_{i})},\ln\frac{\;p_{i,o}}{g(\;p_{i})}\right)$$

where $g(\;p_{i})$ denotes the geometric mean of the four components for unit *i*. The clr transformation maps compositional data into Euclidean space, allowing the application of distance-based clustering algorithms (27). To ensure comparability across components and to prevent variables with greater variance from dominating the clustering solution, the clr-transformed variables were standardized (z-scored) prior to analysis (28).

### 2.4 Cluster analysis

K-means clustering was applied to the standardized clr–transformed dataset. Following clr transformation, the compositional visit data are mapped into Euclidean space, satisfying the distance assumptions required for k-means clustering. This method was selected due to its computational efficiency and its ability to produce centroid-based clusters that are straightforward to interpret and communicate in applied health policy and management contexts. Importantly, cluster centroids can be readily back-transformed to the original compositional scale, allowing each cluster to be described in terms of intuitive service utilization profiles (29).

A range of clustering solutions from two to six clusters was evaluated. The optimal number of clusters (k) was determined using a combination of quantitative and substantive criteria. Quantitatively, the Elbow method was used to examine reductions in within-cluster sum of squares as additional clusters were introduced (30). The corresponding elbow plot is provided in Supplementary Figure 1 and indicates a clear inflection point at k = 3, beyond which reductions in within-cluster variance become marginal. Substantively, the final solution was guided by parsimony and interpretability, favoring configurations that yielded functionally meaningful and policy-relevant typologies of PHC units. To reduce sensitivity to initialization, the k-means algorithm was run with multiple random starting points, and the final solution was selected based on convergence stability and the coherence of the resulting clusters. The final cluster centroids were subsequently back-transformed into compositional form to facilitate interpretation of the dominant visit patterns characterizing each functional typology.

### 2.5 Variables for the association analysis

To explore the determinants of cluster membership, five contextual and organizational variables were constructed. The size of each PHC unit was measured as the natural logarithm of the total number of visits ($ln_{t}$). The organizational type of PHC unit followed the MoH's classification, which distinguishes Urban Health Centers (UHC), Rural Health Centers (RHC), Multipurpose Regional Medical Practices (PPI), Special Regional Medical Practices (EPI), Local Medical Practices (TI), Local Health Units (TOMY), Community Mental Health Centers (KPSY), and a residual “Other” category. Three additional geographical identifiers were included: the regional unit (58 in total), the administrative region (13 in total), and the health region (7 in total) in which each PHC unit operates.

### 2.6 Statistical analysis

All analyses were conducted using IBM SPSS Statistics (version 29). Descriptive statistics were used to summarize PHC unit characteristics and visit composition. Differences in clr-transformed visit proportions across clusters were assessed using one-way analysis of variance (ANOVA). Associations between cluster membership and unit size were examined using one-way ANOVA, while associations with categorical organizational and geographic variables were assessed using Pearson's chi-square tests. Statistical significance was defined as a two-sided *p*-value less than 0.05.

### 2.7 Ethical considerations

The study was based exclusively on aggregated administrative data published by the MoH. No individual-level or identifiable information was used; therefore, ethical approval and informed consent were not required under national and institutional research ethics regulations.

## 3 Results

### 3.1 Descriptive findings

Table 1 presents the distribution of public PHC unit types across the seven health regions of Greece. The data reveal substantial regional heterogeneity in organizational composition. Rural Health Centers (RHC) and Local Health Units (TOMY) constitute the largest shares nationwide, together accounting for more than half of all units, with particularly high concentrations in the sixth health region (Epirus, Western Greece and Peloponnese). Urban Health Centers (UHC) are more prevalent in metropolitan areas such as the first and second health regions, reflecting the urban orientation of these regions. Multipurpose and Special Regional Medical Practices (PPI, EPI) appear more dispersed, serving smaller or remote populations. This distribution underscores the structural diversity of PHC provision, shaped by both demographic and geographic factors.

**Table 1:** Distribution of public PHC unit types across health regions.

Unit type	Health region							
	1st	2nd	3rd	4th	5th	6th	7th	Total
RHC	2	26	16	36	34	67	15	196
TOMY	12	23	21	16	17	36	12	137
UHC	28	24	15	10	10	23	4	114
PPI	1	40	5	9	18	19	6	98
TI	20	10	10	10	6	2	1	59
KPSY	4	6	5	6	4	11	4	40
Other	1	0	2	0	0	0	0	3
EPI	1	1	0	0	0	0	0	2
Total	69	130	74	87	89	158	42	649

RHC, rural health centers; TOMY, local health units; UHC, urban health centers; PPI, multipurpose regional medical practices; TI, local medical practices; KPSY, community mental health centers; EPI, special regional medical practices.

Table 2 summarizes the visit composition and population-adjusted indicators across administrative regions. The findings reveal notable regional disparities in both the composition and utilization of PHC services across Greece. Regular visits constitute the largest share of total visits nationwide (43.5%), followed by emergency (25.3%) and prescription visits (29.1%), reflecting the dominant preventive and chronic care role of PHC units. However, the relative proportions vary by region, with Western Greece showing a particularly high share of emergency visits (32.6%) and the Ionian Islands and Western Macedonia demonstrating greater reliance on prescription visits (47.3% and 44.8%, respectively). Population-Adjusted Visit Rates (PAVR) indicate moderate service use overall, while PHC Unit Density (PHCUD) highlights an uneven spatial distribution of facilities, ranging from 3.5 units per 100,000 inhabitants in Western Greece to 13.1 in Eastern Macedonia & Thrace. Together, these findings suggest persistent regional imbalances in service accessibility, case-mix, and workload within Greece's public PHC system.

**Table 2:** Composition of visits, service availability, and workload among public PHC units by administrative region.

Administrative region	% of total visits by visit type				PAVR					PHCUD
	r	e	p	o	r	e	p	o	total	
Attica	38.7	23.3	37.1	0.9	0.7	0.4	0.6	0.0	1.7	7.7
Central Macedonia	48.7	18.9	28.2	4.3	0.7	0.3	0.4	0.1	1.4	6.7
Thessaly	41.2	22.7	33.4	2.7	0.6	0.3	0.5	0.0	1.4	12.4
Western Greece	51.8	32.6	14.9	0.7	0.5	0.3	0.2	0.0	1.0	3.5
Crete	31.7	26.5	39.9	1.9	0.4	0.4	0.5	0.0	1.3	7.8
Eastern Macedonia & Thrace	36.6	19.8	38.7	4.8	0.7	0.4	0.8	0.1	1.9	13.1
Peloponnese	47.2	25.0	24.3	3.4	0.6	0.3	0.3	0.0	1.2	5.7
Central Greece	39.6	19.5	40.0	0.9	0.7	0.3	0.7	0.0	1.7	11.5
South Aegean	31.1	25.0	41.2	2.8	0.4	0.3	0.5	0.0	1.3	8.7
Epirus	40.7	21.1	35.0	3.2	0.6	0.3	0.5	0.0	1.4	6.6
Western Macedonia	39.7	14.2	44.8	1.3	0.5	0.2	0.5	0.0	1.1	6.5
North Aegean	33.0	21.4	43.4	2.1	0.5	0.3	0.7	0.0	1.6	9.2
Ionian Islands	35.0	15.9	47.3	1.8	0.7	0.3	0.9	0.0	1.9	11.5
Total	43.5	25.3	29.1	2.1	0.6	0.3	0.4	0.0	1.3	6.2

r, regular visits; e, emergency visits; p, prescription visits; o, other visits; PAVR, population-adjusted visit rate; PHCUD, PHC unit density.

### 3.2 Cluster analysis

Table 3 presents the compositional characteristics of service utilization across the three clusters identified through k-means analysis. Cluster 1 (*n* = 308) represents units with a balanced mix of visits, averaging 41.4% regular, 22.5% emergency, and 36.1% prescription visits, with minimal “other” visits. Cluster 2 (*n* = 66) is characterized by a predominance of regular visits (mean 82.4%) and very low prescription visits (0.1%), indicating facilities primarily focused on routine care; this cluster exhibits high variability in emergency (0–74.4%) and other visits (0–29.6%), indicating that, despite its dominant orientation toward routine care, some units also accommodate substantial levels of acute demand. This heterogeneity suggests that the “routine care–focused” classification reflects the prevailing service mix rather than a uniform operational profile across all units. Cluster 3 (*n* = 275) shows a more heterogeneous case-mix, with 44.2% regular, 14.2% emergency, 35.1% prescription, and 6.5% other visits. Overall, the analysis highlights significant heterogeneity in PHC unit function, emphasizing that service composition varies widely between facilities, with some highly specialized and others more balanced in their visit profiles.

**Table 3:** Descriptive statistics of visit composition by cluster.

Cluster	Statistic	% of total visits by visit type			
		r	e	p	o
1	N	308	308	308	308
	Mean	41.4	22.5	36.1	0.0
	Std. Deviation	18.6	15.2	19.7	0.0
	Minimum	0.0	0.0	0.9	0.0
	Maximum	94.4	84.4	94.1	0.2
2	N	66	66	66	66
	Mean	82.4	16.4	0.1	1.2
	Std. Deviation	19.5	19.4	0.1	4.9
	Minimum	25.6	0.0	0.0	0.0
	Maximum	100.0	74.4	0.6	29.6
3	N	275	275	275	275
	Mean	44.2	14.2	35.1	6.5
	Std. Deviation	21.6	14.8	20.1	10.8
	Minimum	0.1	0.0	1.0	0.0
	Maximum	98.2	65.2	99.8	91.1
Total	N	649	649	649	649
	Mean	46.7	18.4	32.0	2.9
	Std. Deviation	23.4	16.0	21.7	7.8
	Minimum	0.0	0.0	0.0	0.0
	Maximum	100.0	84.4	99.8	91.1

r, regular visits; e, emergency visits; p, prescription visits; o, other visits.

The ANOVA results in Table 4 confirm that these compositional differences are statistically significant across clusters for all visit types (*p* < 0.001). The largest F-values were observed for prescription visits (Zscore: clr(p_p_)) and other visits (Zscore: clr(p_o_)), indicating that these components contributed most strongly to between-cluster variance. The pairwise comparisons in Supplementary Table 1 further substantiate this pattern. Almost all differences between clusters are statistically significant (*p* < 0.05), with one noteworthy exception: the emergency visits between clusters 1 and 2 (Zscore: clr(p_e_)) are not significantly different (*p* > 0.05). This finding suggests that while regular, prescription, and “other” visits clearly define the functional boundaries between clusters, emergency activity remains a less decisive feature distinguishing routine-oriented units from generalist ones, possibly reflecting a shared minimum capacity for urgent care across PHC facilities.

**Table 4:** ANOVA for differences in clr-transformed visit proportions across clusters.

Variable	Cluster		Error		F	Sig.
	Mean Square	df	Mean Square	df		
Zscore: clr(p_r_)	128.948	2	0.604	646	213.535	<0.001
Zscore: clr(p_e_)	117.209	2	0.640	646	183.077	<0.001
Zscore: clr(p_p_)	218.925	2	0.325	646	672.975	<0.001
Zscore: clr(p_o_)	234.536	2	0.277	646	846.773	<0.001

df, degrees of freedom; Sig., *p*-value.

### 3.3 Determinants of cluster membership

Regarding PHC unit size, Table 5 reveals that there is a statistically significant association between the size of a PHC unit and its cluster membership (*p* < 0.001). *post-hoc* Tukey's tests (Table 6) indicated that cluster 1 units were significantly larger than those in clusters 2 and 3, while cluster 3 units were significantly larger than those in cluster 2 (all *p* < 0.001). The overall effect size (*η*^2^ = 0.078) denotes a moderate association between cluster membership and unit size.

**Table 5:** Summary statistics for PHC unit size by cluster.

Cluster	N	Mean	SD	95% CI for Mean (Lower–Upper)	ANOVA	Effect Size (*η*^2^)
1	308	9.66	1.05	9.54–9.77	df = 2, 646 F = 27.42 *p* < 0.001	0.078
2	66	8.53	1.69	8.11–8.94		
3	275	9.23	1.19	9.08–9.37		
Total	649	9.36	1.24	9.26–9.45		

SD, standard deviation; CI, confidence interval; df, degrees of freedom; HSD, honestly significant difference.

**Table 6:** Pairwise comparisons of PHC unit size across clusters (tukey HSD *post hoc* tests).

	(I) Cluster Number of Case	(J) Cluster Number of Case	Mean Difference (I-J)	Std. Error	Sig.	95% Confidence Interval	
						Lower Bound	Upper Bound
Tukey HSD	1	2	1.1289*	0.1615	0.000	0.7495	1.5083
		3	0.4304*	0.0988	0.000	0.1984	0.6625
	2	1	−1.1289*	0.1615	0.000	−1.5083	−0.7495
		3	−0.6985*	0.1632	0.000	−1.0819	−0.3151
	3	1	−0.4304*	0.0988	0.000	−0.6625	−0.1984
		2	0.6985*	0.1632	0.000	0.3151	1.0819

* The mean difference is significant at the 0.05 level.

In relation to PHC unit organizational type, Table 7 demonstrates a statistically significant association (*p* < 0.001 for both Chi-square tests), indicating that the functional typologies identified through cluster analysis correspond closely to structural differences among unit types. Specifically, cluster 1 includes primarily Rural Health Centers (RHC: 39.6%) and Local Health Units (TOMY: 20.1%). Cluster 2 is dominated by Local Medical Practices (TI: 30.3%) and Urban Health Centers (UHC: 24.2%), characterized by a strong focus on routine care and low prescription activity. Cluster 3 includes a substantial share of Multipurpose Regional Medical Practices (PPI: 18.9%) and Local Health Units (TOMY: 24.4%), reflecting more diverse or mixed service delivery patterns. Among the unit types with the largest shares nationwide (RHC, UHC, and TOMY), it is notable that while TOMY and UHC are almost evenly distributed between the first and third clusters, the majority of RHC (66.7%) belong to cluster 1, with only 35.2% classified in cluster 3.

**Table 7:** Crosstabulation of cluster membership and PHC unit type.

Cluster	Statistic	Unit type								Total
		EPI	KPSY	Other	PPI	RHC	TI	TOMY	UHC	
1	Count	1	5	2	45	122	22	62	49	308
	% within cluster	0.3	1.6	0.6	14.6	39.6	7.1	20.1	15.9	100.0
	% within unit type	50.0	12.5	66.7	45.9	62.2	37.3	45.3	43.0	47.5
2	Count	1	15	0	1	5	20	8	16	66
	% within cluster	1.5	22.7	0.0	1.5	7.6	30.3	12.1	24.2	100.0
	% within unit type	50.0	37.5	0.0	1.0	2.6	33.9	5.8	14.0	10.2
3	Count	0	20	1	52	69	17	67	49	275
	% within cluster	0.0	7.3	0.4	18.9	25.1	6.2	24.4	17.8	100.0
	% within unit type	0.0	50.0	33.3	53.1	35.2	28.8	48.9	43.0	42.4
Total	Count	2	40	3	98	196	59	137	114	649
	% within cluster	0.3	6.2	0.5	15.1	30.2	9.1	21.1	17.6	100.0
	% within unit type	100.0	100.0	100.0	100.0	100.0	100.0	100.0	100.0	100.0

RHC, rural health centers; TOMY, local health units; UHC, urban health centers; PPI, multipurpose regional medical practices; TI, local medical practices; KPSY, community mental health centers; EPI, special regional medical practices.

Chi-square tests results:.

(i) Pearson Chi-Square: 120.872, degrees of freedom (df): 14, *p* < 0.001.

(ii) Likelihood Ratio: 110.083, df: 14, *p* < 0.001.

**Figure 1:**
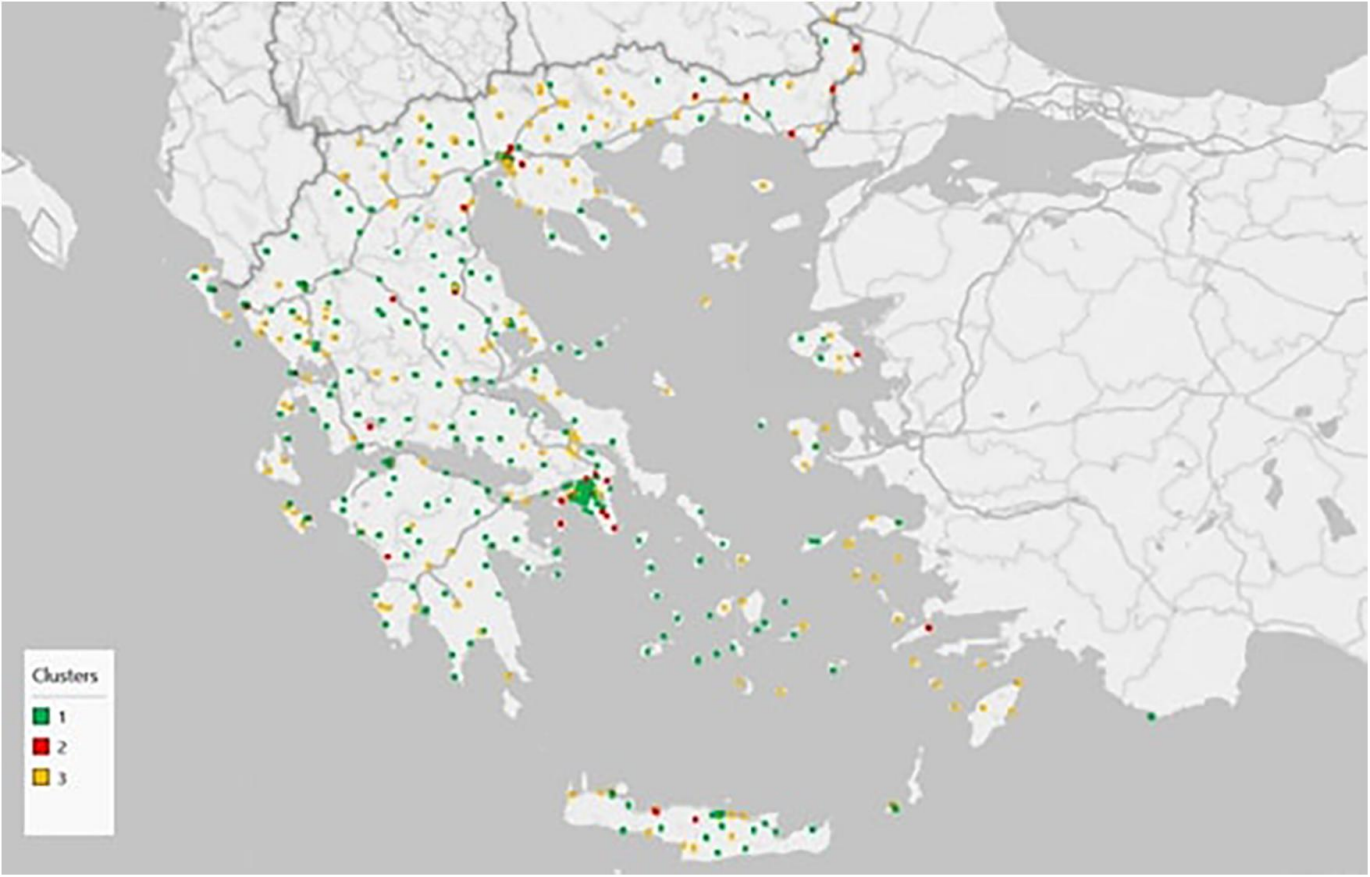
Spatial distribution of PHC unit clusters nationwide in Greece.

**Figure 2:**
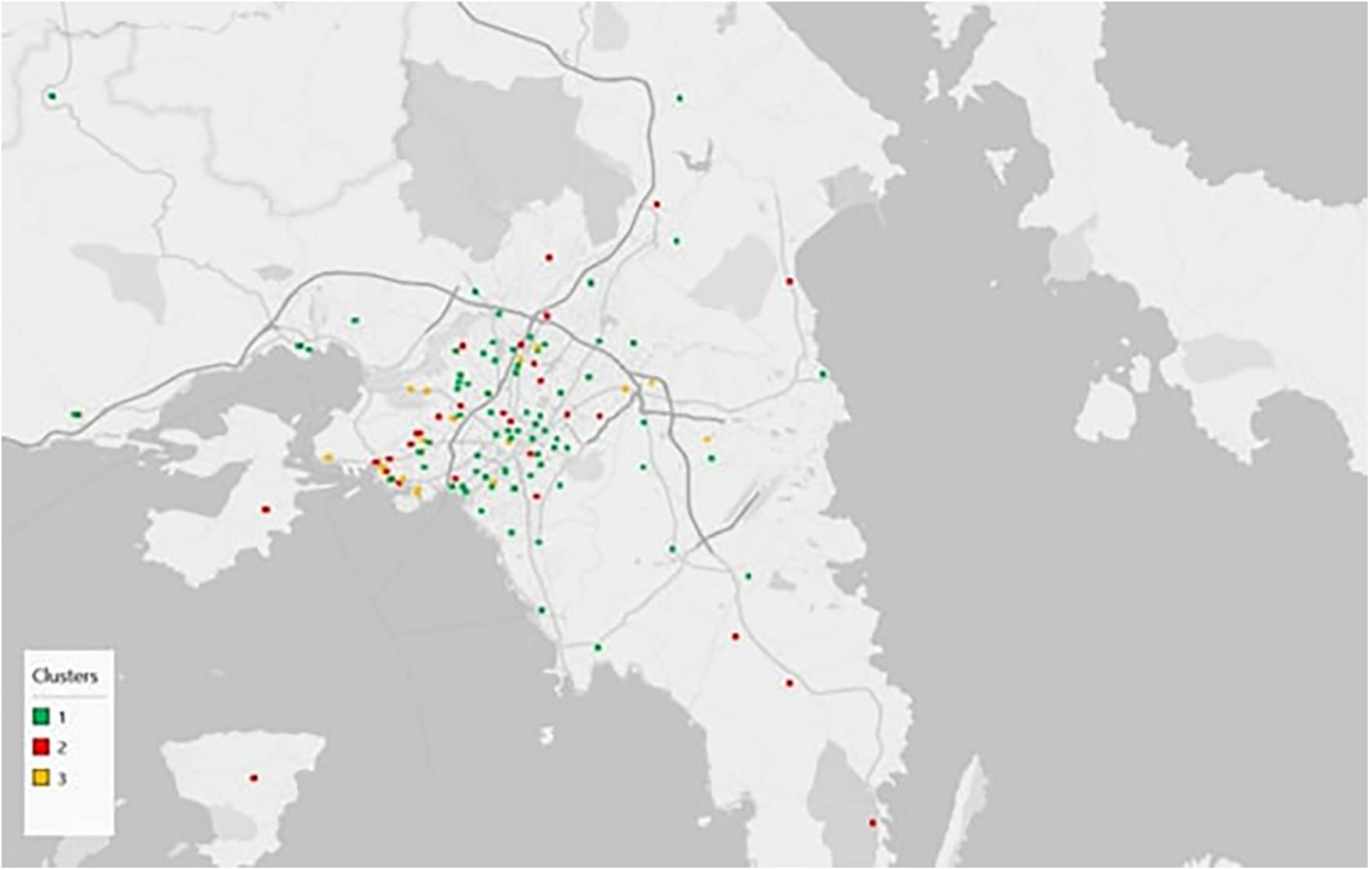
Spatial distribution of PHC unit clusters around Athens.

**Figure 3:**
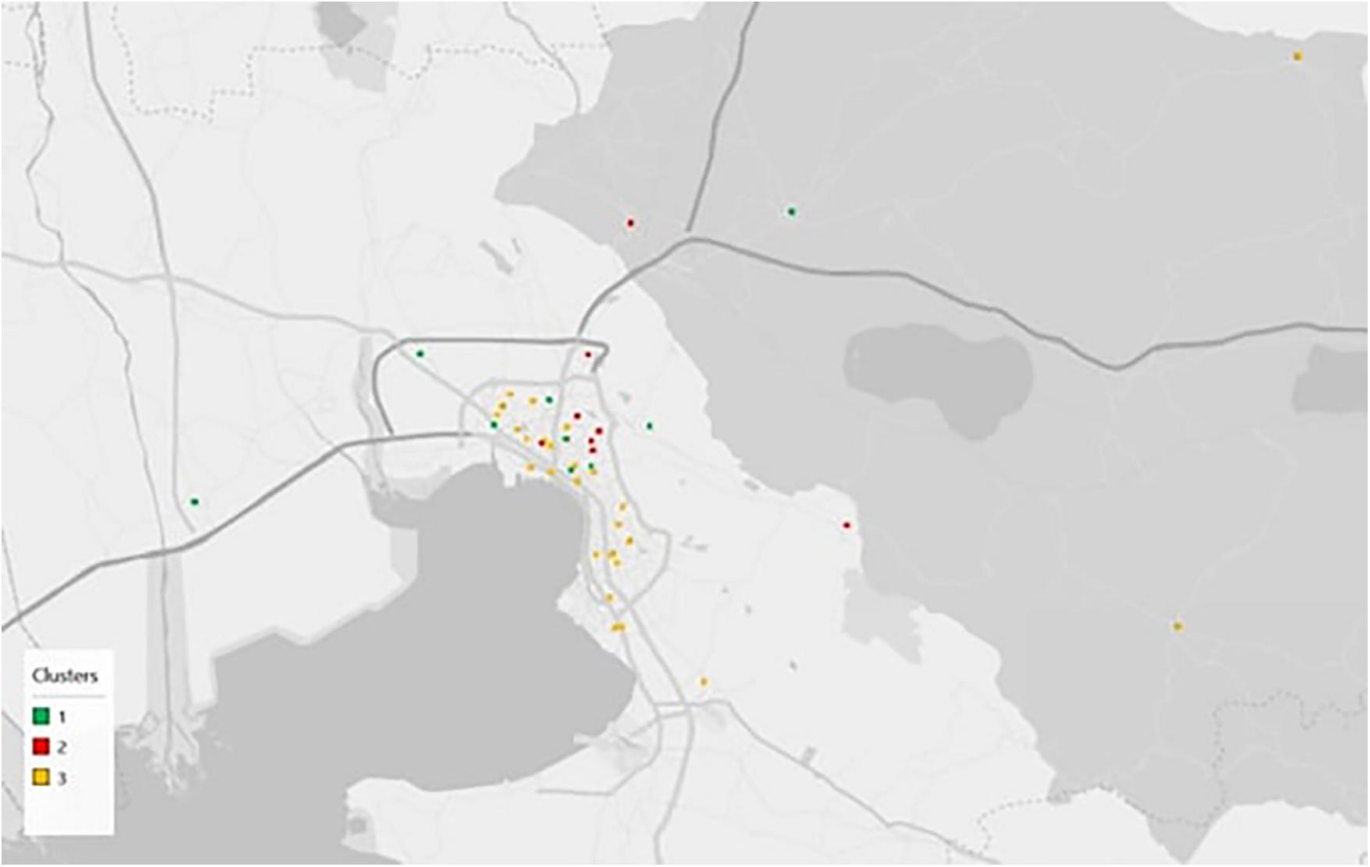
Spatial distribution of PHC unit clusters around thessaloniki.

Subsequently, the analysis examined the spatial distribution of PHC units across Greece. Supplementary Tables 2–4 present the detailed associations between cluster membership and regional unit, administrative region, and health region, respectively, while the corresponding Chi-square tests (Supplementary Tables 5–7) confirm statistically significant relationships in all cases (*p* < 0.001). These results indicate that the functional typologies of PHC units are not randomly distributed but are shaped by distinct geographical and demographic factors. Figure 1 illustrates the nationwide spatial pattern of clusters, showing that cluster 1 units are widely dispersed across the country, particularly in rural mainland areas and on the islands, where they often constitute the backbone of PHC provision. Cluster 2 units appear more sparsely distributed, mainly along major transportation corridors and in selected urban centers. In the Athens metropolitan area (Figure 2), a considerable number of cluster 2 units are located on the periphery of the city, with notable concentrations in the western–southern and northern parts of the region. In contrast, cluster 3 units are concentrated in densely populated areas, primarily around Thessaloniki (Figure 3), the country's co-capital, and secondarily around Athens. Overall, Northern Greece, including Thessaloniki, exhibits a visibly higher proportion of cluster 3 units compared to cluster 1.

## 4 Discussion

This study presents a utilization-based classification of public PHC units in Greece, revealing substantial functional heterogeneity within a formally unified system. Using compositional data analysis and cluster analysis, three distinct functional typologies were identified, differing systematically in service utilization profiles, organizational composition, size, and spatial distribution. These findings demonstrate that PHC units are not functionally homogeneous within administrative or organizational categories, and that observed service delivery patterns are shaped by both structural and contextual factors.

From a health policy and management perspective, the results highlight a critical misalignment between formal organizational labels and actual operational roles. Facilities sharing the same institutional designation may perform markedly different functions, while units with distinct labels may exhibit similar utilization profiles. This misalignment limits the effectiveness of uniform planning, funding, and performance management approaches and underscores the need for function-sensitive PHC governance.

### 4.1 Functional typologies and management implications

The three-cluster solution provides a nuanced understanding of PHC operations across Greece and an empirical basis for functionally differentiated PHC governance, challenging the prevailing assumption of homogeneity in planning, financing, and performance management.

Cluster 1 (“balanced, generalist units”) represents facilities with a relatively even distribution of regular, emergency, and prescription visits and is widely dispersed across rural and insular areas. These units effectively function as comprehensive service providers and often constitute the primary—and in some cases the only—point of access to care for local populations. From a management standpoint, sustaining their role requires stable staffing models, expanded scopes of practice, and funding mechanisms that recognize their broad service mandate. Policy instruments such as enhanced baseline financing, targeted incentives for rural workforce retention, and flexible staffing norms could strengthen their capacity to deliver integrated care, particularly in underserved regions (31). From a system perspective, these units should be explicitly recognized in funding formulas as comprehensive access points, with capitation-based financing adjusted for service breadth to reduce geographic inequities in access.

Cluster 2 (“routine care–focused units”) is characterized by a predominance of regular visits and minimal prescription activity, suggesting a focus on diagnostics, follow-up, preventive services, and chronic disease monitoring. However, the wide range of emergency visit shares observed within this cluster indicates notable within-cluster heterogeneity, suggesting that some units operate beyond a strictly routine care model and retain a variable capacity to respond to acute demand. These units are well positioned to support proactive population health management and continuity of care (8). Policy interventions should prioritize structured chronic care pathways, performance indicators linked to preventive and longitudinal outcomes, and stronger integration with pharmaceutical and referral systems. Aligning reimbursement and evaluation frameworks with continuity-oriented care objectives could enhance the effectiveness and sustainability of these facilities.

Notably, the absence of significant differences in emergency visit shares between clusters 1 and 2 indicates a shared baseline capacity for urgent care across PHC models. This finding has direct policy relevance, as it suggests that strengthening urgent care protocols and triage systems within PHC—rather than expanding hospital emergency capacity—could represent a cost-effective strategy for managing acute demand (32, 33).

Cluster 3 (“mixed, higher-intensity units”) exhibits a heterogeneous case-mix, including substantial proportions of regular, prescription, and “other” visits, and is concentrated in major urban centers such as Athens and Thessaloniki. These units appear to operate as high-throughput service hubs within metropolitan health systems, managing complex demand and a broader range of service activities. From a governance perspective, their role calls for enhanced coordination mechanisms, including interoperable information systems, formalized referral pathways, and prescription oversight frameworks to ensure continuity and safety of care (34). Given their scale and complexity, these units require differentiated governance arrangements, including enhanced managerial autonomy, integrated information systems, and structured coordination with secondary care. They may also serve as focal points for integrated care delivery, supporting referral management, case coordination, and system-level demand regulation.

A notable feature of Cluster 3 is the relatively higher share of “other” visits. As this category reflects a residual administrative classification in the Ministry of Health reporting system, it likely captures a heterogeneous set of activities, including administrative encounters, follow-up visits, referrals, and service-specific tasks not formally categorized as regular, emergency, or prescription visits. The elevated presence of such visits may therefore indicate greater organizational complexity and a broader functional scope, particularly in urban or high-demand settings where PHC units are required to accommodate diverse and non-standardized service needs. At the same time, the interpretive ambiguity of this category highlights limitations in existing reporting systems and underscores the need for more granular classification of PHC activities to better support performance assessment and planning. From a policy perspective, improving the granularity and standardization of visit classification is essential for aligning funding, staffing, and performance evaluation with actual service delivery.

Taken together, the findings support a shift from uniform PHC planning toward a functionally differentiated model, in which resource allocation, workforce policies, and performance frameworks are explicitly aligned with empirically observed service profiles.

### 4.2 Spatial differentiation and regional planning

The pronounced spatial clustering of functional typologies highlights persistent regional imbalances within the Greek PHC system. While the concentration of mixed, high-volume units in urban areas reflects population density and demand, the relative scarcity of certain service profiles in rural and remote regions raises concerns about equitable access. These findings reinforce existing evidence on geographic disparities in healthcare infrastructure and utilization in Greece (20, 22, 35) and internationally (36).

From a policy perspective, this spatial differentiation argues for regionally tailored PHC planning. Health regions dominated by routine-oriented or narrowly focused units may benefit from targeted workforce reinforcement, service integration initiatives, or mobile support teams. Conversely, urban regions with highly diverse service profiles may require stronger coordination and governance mechanisms to manage complexity and prevent fragmentation. Importantly, the functional typologies identified in this study provide a practical tool for aligning regional planning decisions with observed service delivery patterns rather than relying solely on administrative classifications.

### 4.3 Contribution to the literature and limitations

This study advances the literature by empirically demonstrating that PHC systems operating under a formally unified organizational framework may exhibit substantial functional heterogeneity when examined through service utilization patterns. The identification of three distinct typologies highlights how multiple dimensions of care delivery—regular, emergency, prescription, and residual activities—combine in practice to produce differentiated operational roles. This utilization-based perspective moves beyond conventional organization- or function-specific classifications and provides a more accurate representation of how PHC services are actually delivered, with direct implications for evidence-informed management and system design.

Several limitations should be acknowledged. The analysis relied on administrative visit data, which lack information on clinical complexity, patient characteristics, and health outcomes. Consequently, the identified typologies reflect functional differentiation rather than quality or outcome-based distinctions. The “other” visit category represents a heterogeneous residual classification and should be interpreted as indicating broader functional or administrative complexity rather than specific clinical activity. In addition, the cross-sectional design captures a single time point and does not account for temporal changes in PHC roles. Future research incorporating longitudinal data, diagnostic information, and outcome measures could further refine these typologies and assess their implications for quality and efficiency.

## 5 Conclusion

This study provides the first empirical, nationwide classification of public PHC units in Greece based on observed service utilization patterns, demonstrating that the system is far from monolithic. Using a compositional, data-driven approach, three distinct functional typologies were identified: balanced, generalist units that form the backbone of care in rural and remote areas; routine care–focused units oriented toward consultations and chronic disease management; and mixed, high-intensity urban units managing complex and administratively diverse service demands. These findings show that the functional role of PHC units is strongly shaped by unit size, organizational characteristics, and geographic context, revealing pronounced regional imbalances in service provision.

From a policy and management perspective, the results support a shift away from uniform approaches to PHC governance toward models that explicitly account for functional diversity. Aligning financing, workforce planning, and performance frameworks with empirically observed service profiles can enhance system efficiency, strengthen continuity of care, and improve coordination across levels of service delivery.

By grounding PHC reform in observed patterns of service delivery rather than formal institutional labels, this study provides a robust evidence base for more efficient, equitable, and responsive primary care systems. The methodological framework is readily transferable to other health systems using routine administrative data and offers a practical tool for evidence-informed PHC governance.

## Data Availability

The original contributions presented in the study are included in the article/Supplementary Material, further inquiries can be directed to the corresponding author.
