# Supplementary material for: Understanding functional diversity in public primary health care: a cluster analysis of utilization patterns
Source: Front Health Serv. 2026 May 15;6:1782809. doi: 10.3389/frhs.2026.1782809 (PMC13219019; doi:10.3389/frhs.2026.1782809)
Supplement: Supplementary file 1 [file Table1.docx]

Supplementary Material


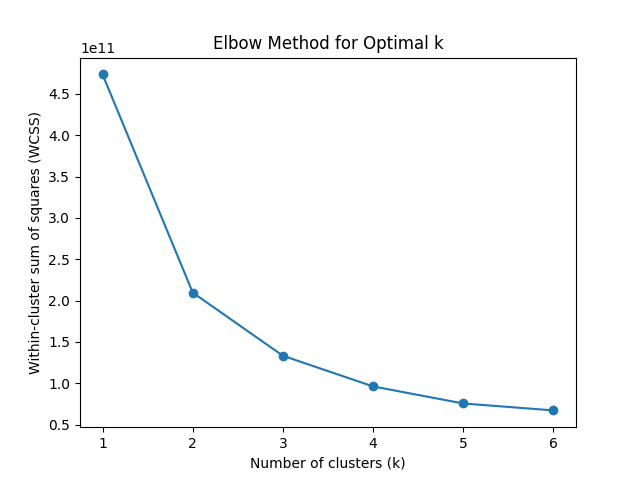


**Supplementary Figure 1**. Elbow plot for determining the optimal number of clusters.

**Supplementary Table 1**. Multiple comparisons of clr-transformed visit proportions between clusters.

| Dependent Variable | | | | Mean Difference (I-J) | Std. Error | Sig. | 95% Confidence Interval | |
| --- | --- | --- | --- | --- | --- | --- | --- | --- |
|  |  |  |  |  |  |  | Lower Bound | Upper Bound |
| Zscore: clr(p_r_) | Tukey HSD | 1 | 2 | -1.6416^*^ | 0.1054 | <0.001 | -1.8893 | -1.3940 |
|  |  |  | 3 | 0.5488^*^ | 0.0645 | <0.001 | 0.3973 | 0.7002 |
|  |  | 2 | 1 | 1.6416^*^ | 0.1054 | <0.001 | 1.3940 | 1.8893 |
|  |  |  | 3 | 2.1904^*^ | 0.1065 | <0.001 | 1.9402 | 2.4406 |
|  |  | 3 | 1 | -0.5488^*^ | 0.0645 | <0.001 | -0.7002 | -0.3973 |
|  |  |  | 2 | -2.1904^*^ | 0.1065 | <0.001 | -2.4406 | -1.9402 |
|  | Bonferroni | 1 | 2 | -1.6416^*^ | 0.1054 | <0.001 | -1.8946 | -1.3887 |
|  |  |  | 3 | 0.5488^*^ | 0.0645 | <0.001 | 0.3940 | 0.7035 |
|  |  | 2 | 1 | 1.6416^*^ | 0.1054 | <0.001 | 1.3887 | 1.8946 |
|  |  |  | 3 | 2.1904^*^ | 0.1065 | <0.001 | 1.9348 | 2.4461 |
|  |  | 3 | 1 | -0.5488^*^ | 0.0645 | <0.001 | -0.7035 | -0.3940 |
|  |  |  | 2 | -2.1904^*^ | 0.1065 | <0.001 | -2.4461 | -1.9348 |
| Zscore: clr(p_e_) | Tukey HSD | 1 | 2 | 0.1765 | 0.1085 | 0.2350 | -0.0784 | 0.4315 |
|  |  |  | 3 | 1.2430^*^ | 0.0664 | <0.001 | 1.0870 | 1.3989 |
|  |  | 2 | 1 | -0.1765 | 0.1085 | 0.2350 | -0.4315 | 0.0784 |
|  |  |  | 3 | 1.0664^*^ | 0.1097 | <0.001 | 0.8088 | 1.3241 |
|  |  | 3 | 1 | -1.2430^*^ | 0.0664 | <0.001 | -1.3989 | -1.0870 |
|  |  |  | 2 | -1.0664^*^ | 0.1097 | <0.001 | -1.3241 | -0.8088 |
|  | Bonferroni | 1 | 2 | 0.1765 | 0.1085 | 0.3130 | -0.0840 | 0.4370 |
|  |  |  | 3 | 1.2430^*^ | 0.0664 | <0.001 | 1.0837 | 1.4023 |
|  |  | 2 | 1 | -0.1765 | 0.1085 | 0.3130 | -0.4370 | 0.0840 |
|  |  |  | 3 | 1.0664^*^ | 0.1097 | <0.001 | 0.8032 | 1.3297 |
|  |  | 3 | 1 | -1.2430^*^ | 0.0664 | <0.001 | -1.4023 | -1.0837 |
|  |  |  | 2 | -1.0664^*^ | 0.1097 | <0.001 | -1.3297 | -0.8032 |
| Zscore: clr(p_p_) | Tukey HSD | 1 | 2 | 2.8381^*^ | 0.0774 | <0.001 | 2.6563 | 3.0198 |
|  |  |  | 3 | 0.4812^*^ | 0.0473 | <0.001 | 0.3700 | 0.5923 |
|  |  | 2 | 1 | -2.8381^*^ | 0.0774 | <0.001 | -3.0198 | -2.6563 |
|  |  |  | 3 | -2.3569^*^ | 0.0782 | <0.001 | -2.5405 | -2.1732 |
|  |  | 3 | 1 | -0.4812^*^ | 0.0473 | <0.001 | -0.5923 | -0.3700 |
|  |  |  | 2 | 2.3569^*^ | 0.0782 | <0.001 | 2.1732 | 2.5405 |
|  | Bonferroni | 1 | 2 | 2.8381^*^ | 0.0774 | <0.001 | 2.6524 | 3.0237 |
|  |  |  | 3 | 0.4811^*^ | 0.0473 | <0.001 | 0.3676 | 0.5947 |
|  |  | 2 | 1 | -2.8381^*^ | 0.0774 | <0.001 | -3.0237 | -2.6524 |
|  |  |  | 3 | -2.3569^*^ | 0.0782 | <0.001 | -2.5445 | -2.1693 |
|  |  | 3 | 1 | -0.4812^*^ | 0.0473 | <0.001 | -0.5947 | -0.3676 |
|  |  |  | 2 | 2.3569^*^ | 0.0782 | <0.001 | 2.1693 | 2.5445 |
| Zscore: clr(p_o_) | Tukey HSD | 1 | 2 | -1.2962^*^ | 0.0714 | <0.001 | -1.4638 | -1.1285 |
|  |  |  | 3 | -1.7727^*^ | 0.0437 | <0.001 | -1.8752 | -1.6701 |
|  |  | 2 | 1 | 1.2962^*^ | 0.0714 | <0.001 | 1.1285 | 1.4638 |
|  |  |  | 3 | -0.4765^*^ | 0.0721 | <0.001 | -0.6460 | -0.3071 |
|  |  | 3 | 1 | 1.7727^*^ | 0.0437 | <0.001 | 1.6701 | 1.8752 |
|  |  |  | 2 | 0.4765^*^ | 0.0721 | <0.001 | 0.3071 | 0.6460 |
|  | Bonferroni | 1 | 2 | -1.2962^*^ | 0.0714 | <0.001 | -1.4675 | -1.1248 |
|  |  |  | 3 | -1.7727^*^ | 0.0437 | <0.001 | -1.8775 | -1.6679 |
|  |  | 2 | 1 | 1.2962^*^ | 0.0714 | <0.001 | 1.1248 | 1.4675 |
|  |  |  | 3 | -0.4765^*^ | 0.0721 | <0.001 | -0.6497 | -0.3034 |
|  |  | 3 | 1 | 1.7727^*^ | 0.0437 | <0.001 | 1.6679 | 1.8775 |
|  |  |  | 2 | 0.4765^*^ | 0.0721 | <0.001 | 0.3034 | 0.6497 |
| Note: “*” means that the mean difference is significant at the 0.05 level. | | | | | | | | |

**Supplementary Table 2**. Crosstabulation of cluster membership and regional unit.

|  | | CNoC | | | | | | | | | Total | | |
| --- | --- | --- | --- | --- | --- | --- | --- | --- | --- | --- | --- | --- | --- |
|  |  | 1 | | | 2 | | | 3 | | |  |  |  |
|  |  | Count | % within CNoC | % within RU | Count | % within CNoC | % within RU | Count | % within CNoC | % within RU | Count | % within CNoC | % within RU |
| RU | Achaia | 14 | 4.5 | 70.0 | 0 | 0.0 | 0.0 | 6 | 2.2 | 30.0 | 20 | 3.1 | 100.0 |
|  | Aitoloakarnania | 12 | 3.9 | 60.0 | 2 | 3.0 | 10.0 | 6 | 2.2 | 30.0 | 20 | 3.1 | 100.0 |
|  | Argolida | 5 | 1.6 | 71.4 | 1 | 1.5 | 14.3 | 1 | 0.4 | 14.3 | 7 | 1.1 | 100.0 |
|  | Arkadia | 5 | 1.6 | 55.6 | 0 | 0.0 | 0.0 | 4 | 1.5 | 44.4 | 9 | 1.4 | 100.0 |
|  | Arta | 3 | 1.0 | 37.5 | 0 | 0.0 | 0.0 | 5 | 1.8 | 62.5 | 8 | 1.2 | 100.0 |
|  | Attica islands | 4 | 1.3 | 57.1 | 2 | 3.0 | 28.6 | 1 | 0.4 | 14.3 | 7 | 1.1 | 100.0 |
|  | Central Athens | 9 | 2.9 | 60.0 | 5 | 7.6 | 33.3 | 1 | 0.4 | 6.7 | 15 | 2.3 | 100.0 |
|  | Chalkidiki | 3 | 1.0 | 30.0 | 0 | 0.0 | 0.0 | 7 | 2.5 | 70.0 | 10 | 1.5 | 100.0 |
|  | Chania | 4 | 1.3 | 40.0 | 1 | 1.5 | 10.0 | 5 | 1.8 | 50.0 | 10 | 1.5 | 100.0 |
|  | Chios | 2 | 0.6 | 25.0 | 1 | 1.5 | 12.5 | 5 | 1.8 | 62.5 | 8 | 1.2 | 100.0 |
|  | Dodekanisos | 5 | 1.6 | 29.4 | 1 | 1.5 | 5.9 | 11 | 4.0 | 64.7 | 17 | 2.6 | 100.0 |
|  | Drama | 1 | 0.3 | 14.3 | 0 | 0.0 | 0.0 | 6 | 2.2 | 85.7 | 7 | 1.1 | 100.0 |
|  | East Attica | 7 | 2.3 | 58.3 | 4 | 6.1 | 33.3 | 1 | 0.4 | 8.3 | 12 | 1.8 | 100.0 |
|  | Evros | 0 | 0.0 | 0.0 | 3 | 4.5 | 25.0 | 9 | 3.3 | 75.0 | 12 | 1.8 | 100.0 |
|  | Evrytania | 0 | 0.0 | 0.0 | 0 | 0.0 | 0.0 | 4 | 1.5 | 100.0 | 4 | 0.6 | 100.0 |
|  | Evvoia | 3 | 1.0 | 25.0 | 0 | 0.0 | 0.0 | 9 | 3.3 | 75.0 | 12 | 1.8 | 100.0 |
|  | Florina | 1 | 0.3 | 33.3 | 0 | 0.0 | 0.0 | 2 | 0.7 | 66.7 | 3 | 0.5 | 100.0 |
|  | Fokida | 2 | 0.6 | 50.0 | 0 | 0.0 | 0.0 | 2 | 0.7 | 50.0 | 4 | 0.6 | 100.0 |
|  | Fthiodita | 9 | 2.9 | 75.0 | 0 | 0.0 | 0.0 | 3 | 1.1 | 25.0 | 12 | 1.8 | 100.0 |
|  | Grevena | 2 | 0.6 | 100.0 | 0 | 0.0 | 0.0 | 0 | 0.0 | 0.0 | 2 | 0.3 | 100.0 |
|  | Ileia | 7 | 2.3 | 77.8 | 1 | 1.5 | 11.1 | 1 | 0.4 | 11.1 | 9 | 1.4 | 100.0 |
|  | Imathia | 1 | 0.3 | 20.0 | 0 | 0.0 | 0.0 | 4 | 1.5 | 80.0 | 5 | 0.8 | 100.0 |
|  | Ioannina | 9 | 2.9 | 64.3 | 0 | 0.0 | 0.0 | 5 | 1.8 | 35.7 | 14 | 2.2 | 100.0 |
|  | Iraklio | 11 | 3.6 | 57.9 | 0 | 0.0 | 0.0 | 8 | 2.9 | 42.1 | 19 | 2.9 | 100.0 |
|  | Karditsa | 6 | 1.9 | 85.7 | 0 | 0.0 | 0.0 | 1 | 0.4 | 14.3 | 7 | 1.1 | 100.0 |
|  | Kastoria | 1 | 0.3 | 50.0 | 0 | 0.0 | 0.0 | 1 | 0.4 | 50.0 | 2 | 0.3 | 100.0 |
|  | Kavala & Thasos | 2 | 0.6 | 25.0 | 0 | 0.0 | 0.0 | 6 | 2.2 | 75.0 | 8 | 1.2 | 100.0 |
|  | Kefallinia & Ithaki | 1 | 0.3 | 20.0 | 0 | 0.0 | 0.0 | 4 | 1.5 | 80.0 | 5 | 0.8 | 100.0 |
|  | Kerkyra | 5 | 1.6 | 55.6 | 1 | 1.5 | 11.1 | 3 | 1.1 | 33.3 | 9 | 1.4 | 100.0 |
|  | Kilkis | 1 | 0.3 | 33.3 | 0 | 0.0 | 0.0 | 2 | 0.7 | 66.7 | 3 | 0.5 | 100.0 |
|  | Korinthia | 6 | 1.9 | 54.5 | 1 | 1.5 | 9.1 | 4 | 1.5 | 36.4 | 11 | 1.7 | 100.0 |
|  | Kozani | 3 | 1.0 | 33.3 | 0 | 0.0 | 0.0 | 6 | 2.2 | 66.7 | 9 | 1.4 | 100.0 |
|  | Kyklades | 19 | 6.2 | 73.1 | 0 | 0.0 | 0.0 | 7 | 2.5 | 26.9 | 26 | 4.0 | 100.0 |
|  | Lakonia | 7 | 2.3 | 63.6 | 0 | 0.0 | 0.0 | 4 | 1.5 | 36.4 | 11 | 1.7 | 100.0 |
|  | Larisa | 11 | 3.6 | 64.7 | 0 | 0.0 | 0.0 | 6 | 2.2 | 35.3 | 17 | 2.6 | 100.0 |
|  | Lasithi | 4 | 1.3 | 80.0 | 0 | 0.0 | 0.0 | 1 | 0.4 | 20.0 | 5 | 0.8 | 100.0 |
|  | Lefkada | 1 | 0.3 | 33.3 | 0 | 0.0 | 0.0 | 2 | 0.7 | 66.7 | 3 | 0.5 | 100.0 |
|  | Lesvos & Limnos | 3 | 1.0 | 33.3 | 1 | 1.5 | 11.1 | 5 | 1.8 | 55.6 | 9 | 1.4 | 100.0 |
|  | Magnisia & Sporades | 7 | 2.3 | 43.8 | 0 | 0.0 | 0.0 | 9 | 3.3 | 56.3 | 16 | 2.5 | 100.0 |
|  | Messinia | 8 | 2.6 | 72.7 | 0 | 0.0 | 0.0 | 3 | 1.1 | 27.3 | 11 | 1.7 | 100.0 |
|  | North Athens | 18 | 5.8 | 66.7 | 8 | 12.1 | 29.6 | 1 | 0.4 | 3.7 | 27 | 4.2 | 100.0 |
|  | Pella | 4 | 1.3 | 44.4 | 0 | 0.0 | 0.0 | 5 | 1.8 | 55.6 | 9 | 1.4 | 100.0 |
|  | Pieria | 2 | 0.6 | 25.0 | 1 | 1.5 | 12.5 | 5 | 1.8 | 62.5 | 8 | 1.2 | 100.0 |
|  | Piraeus | 6 | 1.9 | 24.0 | 8 | 12.1 | 32.0 | 11 | 4.0 | 44.0 | 25 | 3.9 | 100.0 |
|  | Preveza | 3 | 1.0 | 37.5 | 1 | 1.5 | 12.5 | 4 | 1.5 | 50.0 | 8 | 1.2 | 100.0 |
|  | Rethymno | 1 | 0.3 | 12.5 | 3 | 4.5 | 37.5 | 4 | 1.5 | 50.0 | 8 | 1.2 | 100.0 |
|  | Rodopi | 5 | 1.6 | 45.5 | 1 | 1.5 | 9.1 | 5 | 1.8 | 45.5 | 11 | 1.7 | 100.0 |
|  | Samos & Ikaria | 3 | 1.0 | 37.5 | 0 | 0.0 | 0.0 | 5 | 1.8 | 62.5 | 8 | 1.2 | 100.0 |
|  | Serres | 4 | 1.3 | 36.4 | 0 | 0.0 | 0.0 | 7 | 2.5 | 63.6 | 11 | 1.7 | 100.0 |
|  | South Athens | 16 | 5.2 | 94.1 | 1 | 1.5 | 5.9 | 0 | 0.0 | 0.0 | 17 | 2.6 | 100.0 |
|  | Thesprotia | 3 | 1.0 | 42.9 | 1 | 1.5 | 14.3 | 3 | 1.1 | 42.9 | 7 | 1.1 | 100.0 |
|  | Thessaloniki | 8 | 2.6 | 14.5 | 11 | 16.7 | 20.0 | 36 | 13.1 | 65.5 | 55 | 8.5 | 100.0 |
|  | Trikala | 4 | 1.3 | 80.0 | 1 | 1.5 | 20.0 | 0 | 0.0 | 0.0 | 5 | 0.8 | 100.0 |
|  | Voiotia | 6 | 1.9 | 50.0 | 1 | 1.5 | 8.3 | 5 | 1.8 | 41.7 | 12 | 1.8 | 100.0 |
|  | West Athens | 9 | 2.9 | 52.9 | 2 | 3.0 | 11.8 | 6 | 2.2 | 35.3 | 17 | 2.6 | 100.0 |
|  | West Attica | 8 | 2.6 | 72.7 | 0 | 0.0 | 0.0 | 3 | 1.1 | 27.3 | 11 | 1.7 | 100.0 |
|  | Xanthi | 2 | 0.6 | 33.3 | 2 | 3.0 | 33.3 | 2 | 0.7 | 33.3 | 6 | 0.9 | 100.0 |
|  | Zakynthos | 2 | 0.6 | 33.3 | 1 | 1.5 | 16.7 | 3 | 1.1 | 50.0 | 6 | 0.9 | 100.0 |
| Total | | 308 | 100.0 | 47.5 | 66 | 100.0 | 10.2 | 275 | 100.0 | 42.4 | 649 | 100.0 | 100.0 |
| Note: RU: regional unit; CNoC: Cluster Number of Case | | | | | | | | | | | | | |

**Supplementary Table 3**. Crosstabulation of cluster membership and administrative region.

|  | | CNoC | | | | | | | | | Total | | |
| --- | --- | --- | --- | --- | --- | --- | --- | --- | --- | --- | --- | --- | --- |
|  |  | 1 | | | 2 | | | 3 | | |  |  |  |
|  |  | Count | % within CNoC | % within AR | Count | % within CNoC | % within AR | Count | % within CNoC | % within AR | Count | % within CNoC | % within AR |
| AR | Attica | 77 | 25.0 | 58.8 | 30 | 45.5 | 22.9 | 24 | 8.7 | 18.3 | 131 | 20.2 | 100.0 |
|  | Central Greece | 20 | 6.5 | 45.5 | 1 | 1.5 | 2.3 | 23 | 8.4 | 52.3 | 44 | 6.8 | 100.0 |
|  | Central Macedonia | 23 | 7.5 | 22.8 | 12 | 18.2 | 11.9 | 66 | 24.0 | 65.3 | 101 | 15.6 | 100.0 |
|  | Crete | 20 | 6.5 | 47.6 | 4 | 6.1 | 9.5 | 18 | 6.5 | 42.9 | 42 | 6.5 | 100.0 |
|  | Eastern Macedonia and Thrace | 10 | 3.2 | 22.7 | 6 | 9.1 | 13.6 | 28 | 10.2 | 63.6 | 44 | 6.8 | 100.0 |
|  | Epirus | 18 | 5.8 | 48.6 | 2 | 3.0 | 5.4 | 17 | 6.2 | 45.9 | 37 | 5.7 | 100.0 |
|  | Ionian Islands | 9 | 2.9 | 39.1 | 2 | 3.0 | 8.7 | 12 | 4.4 | 52.2 | 23 | 3.5 | 100.0 |
|  | North Aegean | 8 | 2.6 | 32.0 | 2 | 3.0 | 8.0 | 15 | 5.5 | 60.0 | 25 | 3.9 | 100.0 |
|  | Peloponnese | 35 | 11.4 | 64.8 | 3 | 4.5 | 5.6 | 16 | 5.8 | 29.6 | 54 | 8.3 | 100.0 |
|  | South Aegean | 24 | 7.8 | 55.8 | 1 | 1.5 | 2.3 | 18 | 6.5 | 41.9 | 43 | 6.6 | 100.0 |
|  | Thessaly | 24 | 7.8 | 60.0 | 0 | 0.0 | 0.0 | 16 | 5.8 | 40.0 | 40 | 6.2 | 100.0 |
|  | Western Greece | 33 | 10.7 | 67.3 | 3 | 4.5 | 6.1 | 13 | 4.7 | 26.5 | 49 | 7.6 | 100.0 |
|  | Western Macedonia | 7 | 2.3 | 43.8 | 0 | 0.0 | 0.0 | 9 | 3.3 | 56.3 | 16 | 2.5 | 100.0 |
| Total | | 308 | 100.0 | 47.5 | 66 | 100.0 | 10.2 | 275 | 100.0 | 42.4 | 649 | 100.0 | 100.0 |
| Note: AR: administrative region; CNoC: Cluster Number of Case | | | | | | | | | | | | | |

**Supplementary Table 4**. Crosstabulation of cluster membership and health region.

|  | | CNoC | | | | | | | | | Total | | |
| --- | --- | --- | --- | --- | --- | --- | --- | --- | --- | --- | --- | --- | --- |
|  |  | 1 | | | 2 | | | 3 | | |  |  |  |
|  |  | Count | % within CNoC | % within HR | Count | % within CNoC | % within HR | Count | % within CNoC | % within HR | Count | % within CNoC | % within HR |
| HR | 1^st^ | 49 | 15.9 | 71.0 | 18 | 27.3 | 26.1 | 2 | 0.7 | 2.9 | 69 | 10.6 | 100.0 |
|  | 2^nd^ | 60 | 19.5 | 46.2 | 15 | 22.7 | 11.5 | 55 | 20.0 | 42.3 | 130 | 20.0 | 100.0 |
|  | 3^rd^ | 21 | 6.8 | 28.4 | 12 | 18.2 | 16.2 | 41 | 14.9 | 55.4 | 74 | 11.4 | 100.0 |
|  | 4^th^ | 19 | 6.2 | 21.8 | 6 | 9.1 | 6.9 | 62 | 22.5 | 71.3 | 87 | 13.4 | 100.0 |
|  | 5^th^ | 48 | 15.6 | 53.9 | 2 | 3.0 | 2.2 | 39 | 14.2 | 43.8 | 89 | 13.7 | 100.0 |
|  | 6^th^ | 91 | 29.5 | 57.6 | 9 | 13.6 | 5.7 | 58 | 21.1 | 36.7 | 158 | 24.3 | 100.0 |
|  | 7^th^ | 20 | 6.5 | 47.6 | 4 | 6.1 | 9.5 | 18 | 6.5 | 42.9 | 42 | 6.5 | 100.0 |
| Total | | 308 | 100.0 | 47.5 | 66 | 100.0 | 10.2 | 275 | 100.0 | 42.4 | 649 | 100.0 | 100.0 |
| Note: HR: health region; CNoC: Cluster Number of Case | | | | | | | | | | | | | |

**Supplementary Table 5**. Chi-square tests for the association between cluster membership and regional unit.

|  | Value | df | Asymptotic Significance (2-sided) |
| --- | --- | --- | --- |
| Pearson Chi-Square | 234.090^a^ | 114 | <0.001 |
| Likelihood Ratio | 272.496 | 114 | <0.001 |
| N of Valid Cases | 649 |  |  |
| a. 129 cells (74.1%) have expected count less than 5. The minimum expected count is 0.20. | | | |

**Supplementary Table 6**. Chi-square tests for the association between cluster membership and administrative region.

|  | Value | df | Asymptotic Significance (2-sided) |
| --- | --- | --- | --- |
| Pearson Chi-Square | 113.351^a^ | 24 | <0.001 |
| Likelihood Ratio | 122.647 | 24 | <0.001 |
| N of Valid Cases | 649 |  |  |
| a. 10 cells (25.6%) have expected count less than 5. The minimum expected count is 1.63. | | | |

**Supplementary Table 7**. Chi-square tests for the association between cluster membership and health region.

|  | Value | df | Asymptotic Significance (2-sided) |
| --- | --- | --- | --- |
| Pearson Chi-Square | 106.378^a^ | 12 | <0.001 |
| Likelihood Ratio | 121.900 | 12 | <0.001 |
| N of Valid Cases | 649 |  |  |
| a. 1 cells (4.8%) have expected count less than 5. The minimum expected count is 4.27. | | | |
